# Impaired Striatal Akt Signaling Disrupts Dopamine Homeostasis and Increases Feeding

**DOI:** 10.1371/journal.pone.0025169

**Published:** 2011-09-28

**Authors:** Nicole Speed, Christine Saunders, Adeola R. Davis, W. Anthony Owens, Heinrich J. G. Matthies, Sanaz Saadat, Jack P. Kennedy, Roxanne A. Vaughan, Rachael L. Neve, Craig W. Lindsley, Scott J. Russo, Lynette C. Daws, Kevin D. Niswender1, Aurelio Galli

**Affiliations:** 1 Department of Molecular Physiology and Biophysics, School of Medicine, Vanderbilt University, Nashville, Tennessee, United States of America; 2 Department of Pharmacology, School of Medicine, Vanderbilt University, Nashville, Tennessee, United States of America; 3 Center for Molecular Neuroscience, School of Medicine, Vanderbilt University, Nashville, Tennessee, United States of America; 4 Department of Physiology, University of Texas Health Science Center at San Antonio, San Antonio, Texas, United States of America; 5 Department of Biochemistry and Molecular Biology, School of Medicine and Health Science, University of North Dakota, Grand Forks, North Dakota, United States of America; 6 Department of Neuroscience, Mount Sinai Medical Center, New York, New York, United States of America; 7 Department of Brain and Cognitive Sciences, Massachusetts Institute of Technology, Cambridge, Massachusetts, United States of America; 8 Tennessee Valley Healthcare System, Nashville, Tennessee, United States of America; 9 Department of Medicine, School of Medicine, Vanderbilt University, Nashville, Tennessee, United States of America; University of Texas Health Science Center at San Antonio, United States of America

## Abstract

**Background:**

The prevalence of obesity has increased dramatically worldwide. The obesity epidemic begs for novel concepts and therapeutic targets that cohesively address “food-abuse” disorders. We demonstrate a molecular link between impairment of a central kinase (Akt) involved in insulin signaling induced by exposure to a high-fat (HF) diet and dysregulation of higher order circuitry involved in feeding. Dopamine (DA) rich brain structures, such as striatum, provide motivation stimuli for feeding. In these central circuitries, DA dysfunction is posited to contribute to obesity pathogenesis. We identified a mechanistic link between metabolic dysregulation and the maladaptive behaviors that potentiate weight gain. Insulin, a hormone in the periphery, also acts centrally to regulate both homeostatic and reward-based HF feeding. It regulates DA homeostasis, in part, by controlling a key element in DA clearance, the DA transporter (DAT). Upon HF feeding, nigro-striatal neurons rapidly develop insulin signaling deficiencies, causing increased HF calorie intake.

**Methodology/Principal Findings:**

We show that consumption of fat-rich food impairs striatal activation of the insulin-activated signaling kinase, Akt. HF-induced Akt impairment, in turn, reduces DAT cell surface expression and function, thereby decreasing DA homeostasis and amphetamine (AMPH)-induced DA efflux. In addition, HF-mediated dysregulation of Akt signaling impairs DA-related behaviors such as (AMPH)-induced locomotion and increased caloric intake. We restored nigro-striatal Akt phosphorylation using recombinant viral vector expression technology. We observed a rescue of DAT expression in HF fed rats, which was associated with a return of locomotor responses to AMPH and normalization of HF diet-induced hyperphagia.

**Conclusions/Significance:**

Acquired disruption of brain insulin action may confer risk for and/or underlie “food-abuse” disorders and the recalcitrance of obesity. This molecular model, thus, explains how even short-term exposure to “the fast food lifestyle” creates a cycle of disordered eating that cements pathological changes in DA signaling leading to weight gain and obesity.

## Introduction

Insulin is an essential hormone with pleiotropic effects in multiple tissues and has an important function in the regulation of plasma glucose levels in peripheral tissues. In the CNS, insulin relays homeostatic signals regarding the status of peripheral energy metabolism. The downstream cellular effects of insulin receptor signaling include activation of phosphoinositide 3-kinases (PI3K), which has been demonstrated to play a key role in insulin regulation of feeding [1] and many other insulin-controlled processes. PI3K phosphorylates the D-3 position of phosphoinositides to generate PI(3,4,5)P_3_ (PIP_3_) [1], [2], ultimately activating protein kinase B (Akt) at the plasma membrane. Akt is a key element in insulin and growth factor signaling that controls several cellular functions, including cell growth and apoptosis [3]. In the CNS, Akt is involved in feeding behavior [4] as well as in the regulation of dopamine (DA) signaling [5], [6] and DA homeostasis [7], [8], [9], [10].

The DA transporter (DAT) controls the strength and duration of DA neurotransmission by mediating synaptic DA clearance *via* uptake of DA. DAT is, therefore, a critical regulator of DA homeostasis. We and others have linked CNS insulin action to DA homeostasis by demonstrating its ability to regulate DAT trafficking and function [11], [12], [13]. Tyrosine kinase inhibitors, which block the receptors activated by insulin and insulin-like growth factors, reduce DA clearance by decreasing DAT surface expression [14]. Inhibition of PI3K and Akt also dramatically reduces DA clearance and surface expression of DAT [7], [15], [16]. Thus, evidence indicates that insulin signaling maintains appropriate dopaminergic tone by regulating DAT function. Consistent with this novel role in insulin signaling, insulin receptors (IRs) are expressed in midbrain DA neurons [17].

DA is an important modulator of complex behaviors, including motivation to eat and the pleasure derived from feeding [18], [19]. A convincing role for DA in feeding behavior was demonstrated both by studies revealing profound feeding defects in DA deficient animals [18], [19] and by impaired DA signaling in obesity [20], [21], [22]. Functional imaging studies in humans show that upon eating a palatable meal, DA rich brain regions, such as the striatum, increase in activity [20]. This increase in activity is blunted in obesity in a body mass index (BMI)-dependent manner, an effect accentuated in individuals with a genetic polymorphism associated with reduced DA D2 receptor (D2R) expression [20]. This suggests that dysregulation of DA neurotransmission occurs in obese individuals, and that the genetic trait for impaired DA signaling may predispose to obesity.

In obese rats, DA turnover in the subcortical DA circuitry is impaired [23], and mRNA expression for DAT, DA receptor, and tyrosine hydroxylase (TH), all key elements controlling DA neurotransmission, are reduced [24], [25]. Diet-induced obese (DIO) rats also exhibit impaired electrically stimulated DA release in slices from both the ventral and dorsal striatum [26], further supporting the hypothesis that obesity reduces DA neurotransmission in these striatal regions. Consistent with this striatal DA hypofunction, Johnson and Kenny [22] observed that striatal D2R were also downregulated in obese rats leading to reward hypofunction. These data raise the possibility that impaired DA signaling overrides homeostatic signals that would otherwise constrain feeding, thereby initiating a cycle of over-eating (or non-homeostatic consumption), worsening obesity in affected individuals [12].

Taken together, it is increasingly apparent that DA neurotransmission plays an important role in determining eating patterns. However, it is unclear how increased caloric intake and/or high-fat feeding disrupts DA homeostasis, potentially contributing to obesity and whether restoration of normal DA neurotransmission normalizes caloric intake. Here, we show that in a well characterized model of high-fat (HF) feeding [27], impairment of DA clearance and DA-associated behaviors results from altered DAT trafficking. The molecular mechanism(s) underlying impaired DAT function are striatal insulin resistance and impaired Akt activity. Restoration of striatal Akt activity with viral genetic rescue normalizes DAT expression, DA-associated behaviors and caloric intake. Thus, these data are among the first to provide evidence linking striatal insulin resistance with striatal DA hypofunction and obesity.

## Results

### Diet-induced obesity impairs Akt signaling in striatum and substantia nigra

Central DA signaling pathways are increasingly recognized in the regulation of feeding behavior [18], [19]. Both animal and human studies have consistently demonstrated differences in DA signaling between lean subjects versus those with a spectrum of eating disorders [18], [19], [21], [26]. These changes localize largely to the ventral and dorsal striatum [19], implicating non-hypothalamic or “non-homeostatic” dopaminergic mechanisms in pathological eating and obesity [26].

We and others have previously demonstrated that insulin signaling *via* the downstream kinase, Akt, regulates DA homeostasis in the CNS. Thus, we hypothesized that striatal DA dysfunction induced by an obesogenic HF diet could arise from impairment in central insulin signaling (or other tyrosine kinase signals) through Akt [12]. To test this, we utilized a rat HF feeding DIO model that is characterized by hypothalamic neuronal insulin resistance [27], [28]. Rats were fed a 60% lard-based, HF diet for 28 days, and controls were fed a micro-nutrient matched 10% low-fat diet (LF). Throughout the 28 day period, HF rats consumed significantly more calories than LF animals (Fig. S1A; *p<0.05 by Student's t-test), and gained significantly more weight than their LF counterparts (Fig. S1B; *p<0.05 by Student's t-test). While blood glucose levels were not different, plasma insulin levels were significantly elevated in the HF animals, indicating the presence of insulin resistance (Fig. S1C, S1D; *p<0.05 by Student's t-test). Our model, thus, parallels other DIO models [27].

**Figure 1:**
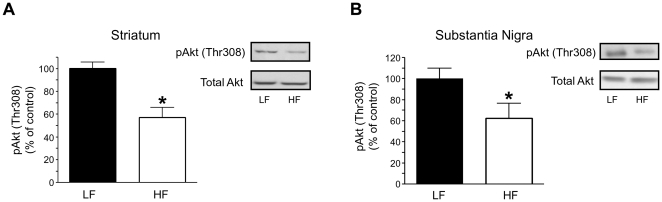
DIO induces a decrease in pAkt(Thr308) in the striatum and substantia nigra. Tissue from the striatum (A) and substania nigra (B) was analyzed by immunoblotting for levels of pAkt (Thr308) and total Akt. Data are expressed as a percent of LF control rats (LF, n = 6; HF, n = 7; *p<0.05 by Student t-test;). Data are represented as mean ± S.E.M.

Akt is activated by phosphorylation at Thr308 in response to insulin, which is commonly utilized as a marker of insulin action. To determine whether DIO results in impaired striatal and/or nigral Akt function, we quantified phosphorylation of Akt at position Thr308 (pAkt) in immunoblots from rapidly dissected striatal and nigral brain extracts. HF DIO resulted in a significant reduction of striatal pAkt compared to LF feeding in rats (Fig. 1A; *p<0.01 by Student's t-test). Similar results were obtained in the substantia nigra (Fig. 1B; *p<0.05 by Student's t-test), whereas total Akt levels were unchanged by HF DIO (Fig. 1A, 1B). These data demonstrate that HF feeding for 28 days leads to DIO as well as reduced nigra-striatal Akt activation.

### HF feeding reduces DA clearance *in vivo*

**Figure 2:**
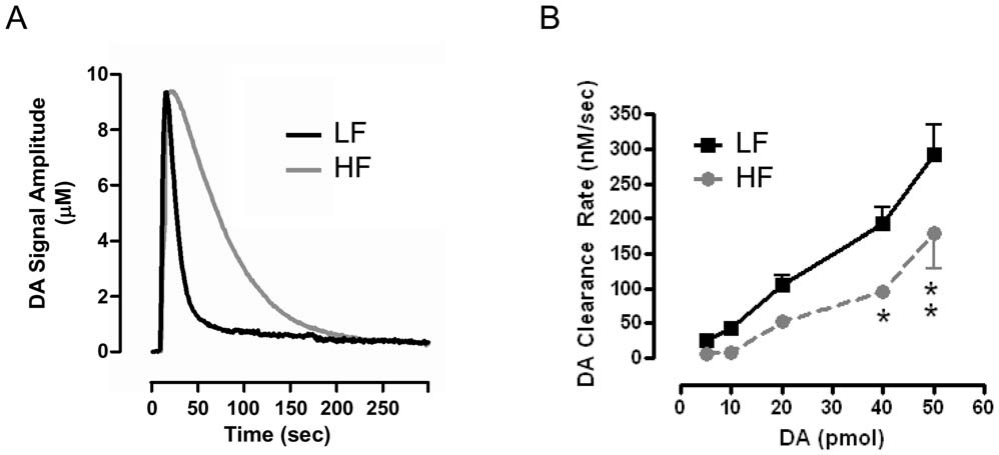
DA clearance rate is reduced in HF DIO rats. (A) Representative oxidation currents produced by intrastriatal application of exogenous DA, and converted to a micromolar concentration using a calibration factor. (B) DA clearance rates were obtained by pressure ejecting increasing pmol amounts of DA in the striatum of anesthetized LF- and HF-fed rats. (n = 4/group, two-way, repeated measures ANOVA, F_1,24_ = 6.843; *p<0.05; **p<0.01 by Bonferroni post-hoc analysis). Data are expressed as mean ± S.E.M.

Evidence in humans and animal models suggests that dysfunction in striatal DA homeostasis has a pathophysiological role in obesity [18], [19], [21], [29]. Thus, we determined whether changes in striatal insulin function due to HF feeding leads to impaired DA clearance *in vivo* using high speed chronoamperometry (HSCA). A carbon fiber recording electrode was lowered into the striatum of anesthetized rats and DA pulsed to measure its clearance time. The kinetic profile of DA clearance after application of DA (50 pmol) demonstrated dramatically delayed clearance in HF compared to LF rats (Fig. 2A). There was no effect on the signal amplitude or on the rise time of the signal attained for a given amount of exogenously applied DA (Fig. 2A), which indicates that HF feeding did not change the rate of DA diffusion through the extracellular matrix. HF feeding significantly reduced the rate of DA clearance compared to LF animals across concentrations of pulsed DA (Fig. 2B, by two-way, repeated measures ANOVA, F_1,24_ = 6.843; *p<0.05, **p<0.01 by Bonferroni post-hoc analysis). Thus, HF feeding, which decreases striatal Akt activity, also decreases striatal DAT function *in vivo*. The implication is that impaired striatal Akt activation induced by an obesogenic diet may dysregulate DA homeostasis by diminishing DAT function.

### HF feeding reduces DAT cell surface expression

**Figure 3:**
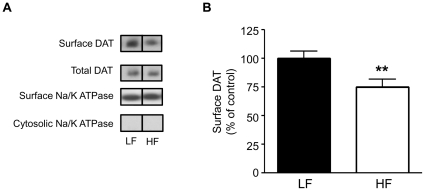
DAT cell surface expression is reduced in the striatum of HF DIO rats. (A) Representative immunoblots of biotinylated (surface) and total DAT proteins from LF and HF rats. The Na/K ATPase was used as a plasma membrane/loading control. (B) Quantification of DAT immunoreactivity. DAT surface levels were normalized to the total amount of DAT and expressed as a percent of LF control. (LF, n = 4; HF, n = 5; **p<0.01 by Students t-test,). Data are represented as mean ± S.E.M.

DA clearance is affected by the number of transporters at the cell surface and by their catalytic function. Therefore, the reduction in DAT activity observed on a HF diet may mechanistically result from a reduction in DAT surface expression and/or activity. We next determined whether HF feeding causes a decrease in striatal DAT cell surface localization utilizing a biotinylation assay. Striatal slices (300 µm) from HF and LF rats were biotinylated and assessed for differences in DAT distribution by immunoblotting. Representative immunoblots of both biotinylated (surface) and total cell fractions from HF and LF rats (Fig. 3A) reveal that cell surface DAT expression was significantly reduced (Fig. 3B, *p<0.05 by Student's t-test). Total levels of DAT remained unchanged (Fig. 3A) and surface levels of the Na/K ATPase, a protein found predominantly at the plasma membrane, were also unchanged. Thus, HF feeding specifically impairs cellular trafficking of DAT. These data suggest a novel molecular mechanism in which DIO, by impairing striatal Akt signaling, alters DA homeostasis (Fig. 2), namely DAT trafficking (Fig. 3).

### Pharmacological inhibition of Akt reduces DAT activity

**Figure 4:**
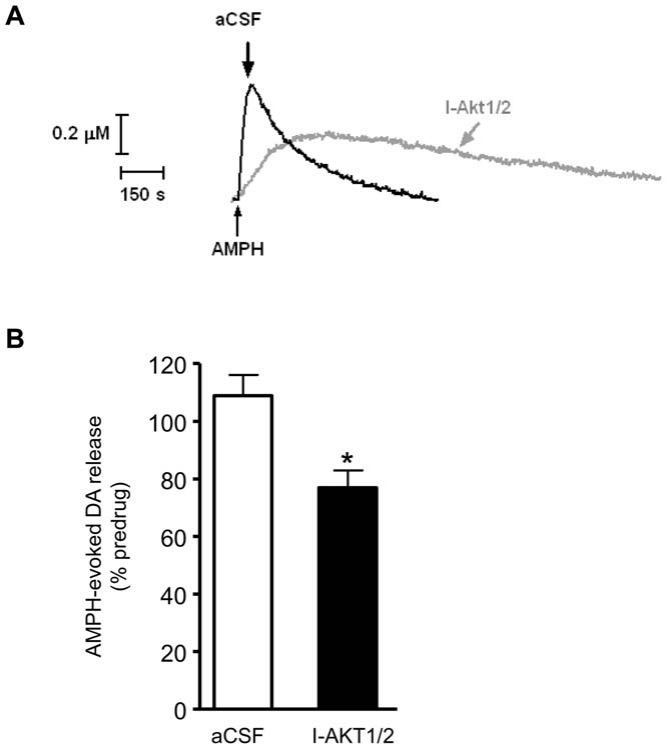
Inhibition of Akt reduces DAT-mediated reverse transport of DA. (A) Representative recordings of striatal extracellular DA release after microinjection of AMPH (400 µM/125 nl) as measured by HSCA. Traces were obtained 45 minutes after microinjection of the inhibitor I-Akt1/2 (1 mM/125 nl, grey trace) or vehicle control (black trace). (B) Quantification of the peak DA release after microinjection of AMPH. (control, n = 9; I-Akt1/2, n = 7; t_14_ = 3.49, *p<0.01, Student's t-test. ). Data are represented as mean ± S.E.M.

To confirm the mechanistic link between Akt activity and DAT expression/function, we inhibited Akt pharmacologically *in vivo*. We utilized amphetamine (AMPH), a substrate of DAT that reverses its transport cycle to cause DA efflux and increase extracellular DA levels. Here, AMPH was utilized to probe DAT activity in order to relate these findings to other AMPH-associated behaviors, such as locomotion (see below). HSCA was used to monitor AMPH-induced DA efflux in striatum while inhibiting Akt function with an allosteric inhibitor of Akt (I-Akt1/2), which targets both Akt1 and Akt2 isoforms [30] and reduces DAT surface expression [16]. The inhibitor, AMPH, and vehicle control (aCSF) were intrastriatally applied with a calibrated micropipette positioned adjacent to the recording electrode. AMPH was applied to obtain a baseline for DA efflux. Then, I-Akt1/2 or aCSF were pressure-ejected in the striatum. After an additional 45 minutes, AMPH was again pressure-ejected and recording continued (Fig. 4A). The release of DA was calculated as a percent of the AMPH-induced amperometric signal (peak) during baseline recording (absence of inhibitor). Inhibition of Akt led to a significant reduction in the ability of AMPH to cause DA efflux (Fig. 4A, B, *p<0.01 by Student's t-test). This supports our hypothesis that changes in Akt function induced either pharmacologically or by HF-feeding modify striatal DAT function and DA homeostasis.

### HF feeding impairs AMPH-induced locomotion

**Figure 5:**
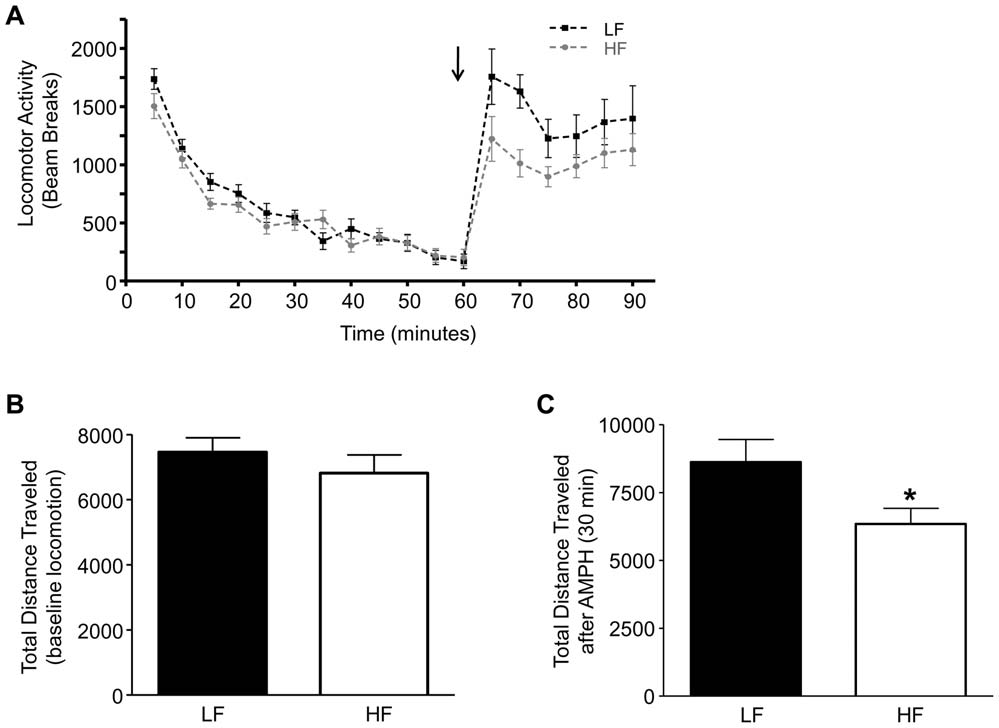
AMPH-induced locomotor activity is reduced in HF DIO rats. Locomotor activity was assessed in HF and LF rats before and after an i.p. injection of AMPH (1.78 mg/kg). (A) Locomotor activity measured by beam breaks over time. Each data point represents 5 minutes of recording bins. The arrow indicates administration of AMPH at 60 minutes. (B) Total distance traveled by HF and LF rats measured during the first 60 minutes (before AMPH; p≥0.05 by Student's t-test, n = 12). (C) Total distance traveled measured in HF and LF rats in a 30 min time period after AMPH injection (*p<0.05 by Students t-test, n = 12). Data are represented as mean ± S.E.M.

Locomotion is a DA-associated behavior and AMPH, which increases locomotion, requires proper subcortical DA homeostasis [31]. Therefore, to establish whether HF-induced reductions in striatal Akt function and DAT activity also impair integrated striatal DA function *in vivo*, we measured AMPH-induced locomotion in HF and LF fed animals. Movement was assessed by the total beam breaks (activity counts) in a 5 min interval and plotted over time (Fig. 5A). After 60 min of baseline activity, AMPH (1.78 mg/kg, i.p.) was administered (arrow) and monitoring continued for 30 min. Baseline locomotor activity (0 to 60 min) was unchanged by HF feeding (Fig. 5B). In contrast, AMPH-induced locomotion was reduced by HF feeding (Fig. 5C, *p<0.05 by Student's t-test). To further determine whether HF feeding decreases AMPH-induced locomotion additionally by altering DA receptor signaling, locomotion was examined after administration of apomorphine (2.0 mg/kg, i.p.), a DA receptor agonist. Unlike AMPH, apomorphine-induced locomotor activity (total distance traveled) did not significantly differ between LF and HF rats over a 60 min period (data not shown). These data are consistent with the idea that the reduction in AMPH-induced locomotion induced by HF feeding is mediated by a decrease in DAT plasma membrane expression (Fig. 3), not impaired DA receptor signaling.

### Virally mediated IRS2 expression rescues Akt activity in the substantia nigra

**Figure 6:**
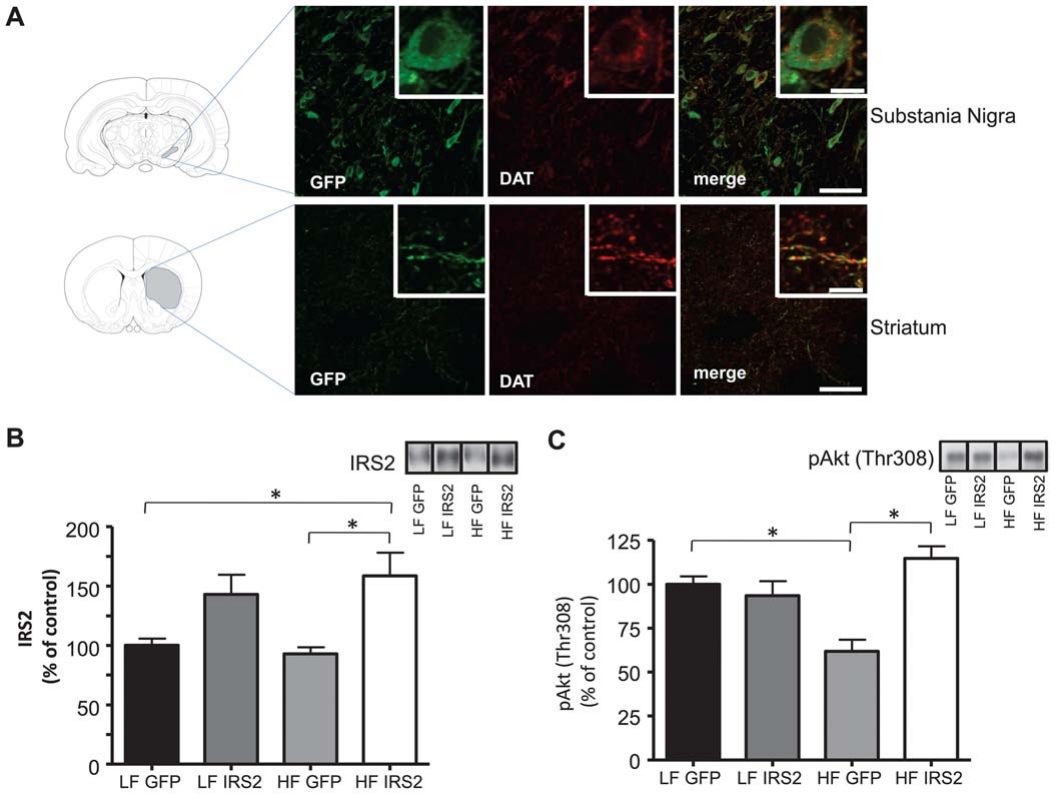
Viral-mediated expression of IRS2 in HF rats restores pAkt levels. Viral injection was performed as described in Methods. (A) Dopaminergic neurons from the substantia nigra (top row) and projection terminals in the striatum (bottom row) were probed for GFP and DAT after injection of HSV-GFP into the substantia nigra. The first column (green) represents GFP, the second column (red) DAT, and the third column represents the merging of these channels together to demonstrate co-expression in the same cell type (scale bars: 10 µm inset; 50 µm main panels; representative of n = 6 for LF; n = 8 for HF). (B) Representative immunoblot and quantification of IRS2 levels in the substania nigra after injection of either HSV-GFP or HSV-IRS2 in LF and HF animals (n = 4 per each group, *p<0.05 by one-way ANOVA, followed by Bonferroni post hoc test). (C) Representative immunoblot and quantification of pAkt (Thr308) levels in the substania nigra after injection of HSV-GFP or HSV-IRS2 in LF and HF animals. (LF, n = 3; HF, n = 4; *p<0.05 by one-way ANOVA, followed by Bonferroni post hoc test). All data are represented as mean ± S.E.M.

To further support our hypothesis that HF DIO impairs DAT function mechanistically by impairing Akt activity, we employed viral gene delivery technology to increase expression of insulin receptor substrate 2 (IRS2), a cytosolic protein upstream of Akt whose activation increases Akt function [32]. A recombinant herpes simplex virus (HSV) encoding IRS2 (HSV-IRS2) or an HSV encoding GFP only (HSV-GFP) as a control were injected into the substantia nigra of both HF and LF animals. This virus has been previously demonstrated to generate efficient gene expression in dopaminergic regions of the rodent brain [33]. We confirmed accurate injection coordinates and efficient viral neuronal transduction by examining GFP expression in the cell bodies of the nigral dopaminergic neurons and in their terminal projections to the striatum (Fig. 6A). Sections were also immunostained against DAT to mark dopaminergic neurons. We observed strong co-localization of GFP with DAT in nigral cell bodies and in striatal terminals (Fig. 6A). Since IRS2 is upstream of Akt, we anticipated that overexpression of IRS2 would restore Akt activation in HF animals, as has been observed in other models [32], [33]. A significant increase in IRS2 protein level was observed in the substantia nigra of HF HSV-IRS2 injected animals compared to GFP controls (HF GFP) (Fig. 6B, *p<0.05; one-way ANOVA followed by Bonferroni post hoc test). Importantly, IRS2 overexpression rescued the impairment in basal Akt phosphorylation observed in HF-fed rats (Fig. 6C; compare HF GFP with HF IRS2, *p<0.05, one-way ANOVA followed by Bonferroni post hoc test). These data demonstrate that nigral IRS2 overexpression restores Akt function in HF DIO animals.

### Nigral IRS2 injection rescues DAT cell surface expression in striatum and DA associated behaviors

**Figure 7:**
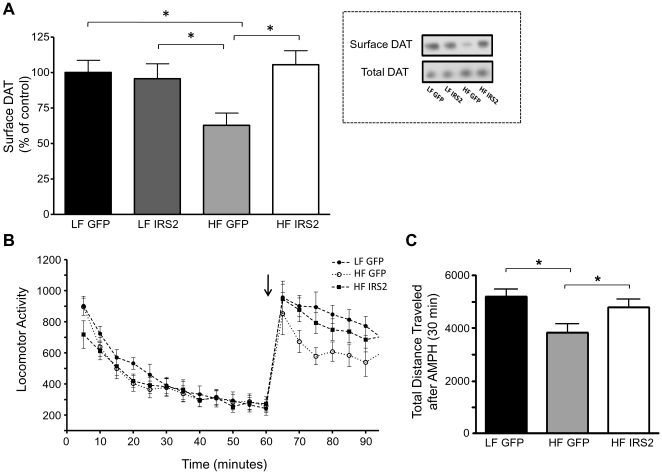
Viral-mediated expression of IRS2 restores surface expression of DAT and AMPH-induced locomotor activity in HF rats. (A) Inset: Representative immunoblots of biotinylated (surface) and total DAT obtained from LF and HF rats injected either with HSV-GFP (LF GFP or HF GFP) or HSV-IRS2 (LF IRS2 or HF IRS2). DAT surface levels were normalized to total and expressed as a percentage of LF, HSV-GFP injected rats (n = 6 per group; *p<0.05 by one-way ANOVA, followed by Bonferroni post hoc test). (B) Locomotor activity measured by beam breaks over time, with AMPH (arrow) given at 60 minutes (1.78 mg/kg, i.p.) to HF rats injected either with HSV-IRS2 (HF IRS2) or HSV-GFP (HF GFP), and LF rats injected with HSV-GFP (LF GFP). (C) Total distance traveled in a 30 min time period after injection of AMPH in HF IRS2, HF GFP and LF GFP injected rats (LF GFP, n = 13; HF GFP, n = 12; HF IRS2, n = 13; *p<0.05 by one-way ANOVA, followed by Newman-Keuls multiple comparison test). Data are represented as mean ± S.E.M.

To mechanistically test whether DIO-induced brain Akt impairment contributes to DA dysfunction, we next investigated whether genetic IRS2 rescue restores DAT function and associated behaviors. In a similar design, LF and HF animals were injected in the substantia nigra with either HSV-GFP or HSV-IRS2 and DAT membrane expression quantified in striatal slices by biotinylation (Fig. 7A, inset). Whereas nigral injection of HSV-IRS2 did not increase striatal DAT cell surface expression in LF animals (compared to GFP injected controls), viral IRS2 overexpression in HF animals clearly rescued DAT plasma membrane expression to the level detected in LF controls (Fig. 7A; HF IRS2 vs. HF GFP, *p<0.05 by one-way ANOVA, followed by Bonferroni post hoc test). Thus, genetically rescuing impaired brain Akt signaling (IRS2) normalized DAT surface expression. Next, we determined whether this rescue could reverse the impaired AMPH-induced locomotion observed in HF animals. Three days after injection with either HSV-GFP or HSV-IRS2 into the substantia nigra of LF and HF rats, locomotion responses were measured after injection of 1.78 mg/kg (i.p.) of AMPH (Fig. 7B). HF HSV-GFP injected rats showed reduced cumulative locomotion 30 minutes after the AMPH injection compared to LF HSV-GFP injected rats (Fig. 7C, *p<0.05 by one-way ANOVA followed by Bonferroni post hoc test). HSV-IRS2 injection, however, rescued this locomotor defect, increasing cumulative activity to a level that was not significantly different from LF rats injected with HSV-GFP (Fig. 7C). Thus, we observed that IRS2 rescue of Akt activity restores deficits in both cell surface expression of DAT and DA-associated behavior in HF rats.

### Nigral IRS2 injection restores caloric intake to the levels of LF fed animals

**Figure 8:**
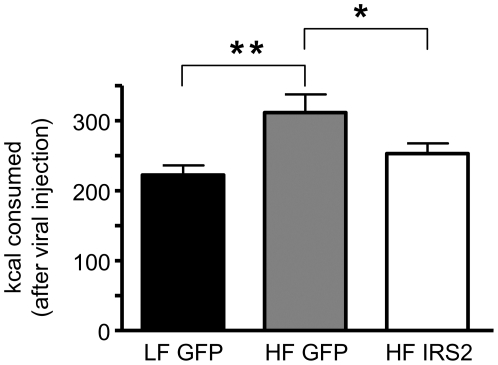
HF feeding results in more calories consumed, an effect reversed by viral injection of IRS2. Caloric consumption of rats used in experiments in Fig. 7 was calculated over the three day, post-injection period. (LF GFP, n = 11; HF GFP, n = 18; HF IRS2, n = 13; **p<0.01; *p<0.05 by one-way ANOVA, followed by Newman-Keuls multiple comparison test). Data represented are the mean ± S.E.M.

Studies in both animals and humans, including our own data, support the concept that HF DIO leads to significant effects on brain monoamine homeostasis and that this may contribute to hyperphagia and weight gain [12]. Our data suggest that neuronal Akt impairment is one mechanism underlying brain DA dysfunction. Thus, we sought to determine if normalization of nigra-striatal Akt function by viral rescue (HSV-IRS2 injection in substantia nigra) in HF animals rescues the hyperphagia observed on a HF diet. Using the same design as in Fig. 7, HSV-GFP injected HF rats consumed significantly more calories over three days compared to LF HSV-GFP rats (Fig. 8). HSV-IRS2 injection in HF rats normalized caloric intake to the level of HSV-GFP injected LF rats (Fig. 8). These data provide a mechanistic framework for linking brain insulin resistance induced by DIO and impaired Akt activity to striatal DA dysfunction and increased caloric intake.

## Discussion

The prevalence of obesity and related disorders such as diabetes has increased worldwide, despite a significant effort to therapeutically target homeostatic mechanisms. The failure of these efforts suggests the existence of additional, non-homeostatic mechanisms that mediate feeding behavior [18]. At the cellular and molecular level, “reward” (non-homeostatic) circuits originating in DA-rich brain structures are increasingly understood to provide motivation and reward stimuli for feeding [18]. The nigrostriatal DA pathway is involved in motivation to engage in feeding behaviors [19], [34], highlighting DA signaling as an important mediator of feeding and therefore, potentially in the development of obesity [29].

In the current environment of fast-food and increasing health issues related to obesity, understanding the effects of high-fat diets on DA systems have become the focus of intense research. Compelling data obtained from rats maintained on a “cafeteria style” diet including highly palatable, high caloric density foods such as meats, cheeses, cookies, sweetened condensed milk, etc., demonstrates defective central DA release [26]. In obese rats, striatal D2R are also downregulated, leading to reward hypofunction [22]. Interestingly, reward hypofunction is thought to underlie mechanisms of illicit substance-use, suggesting the fascinating possibility that obesity and drug-addiction share a common neuronal hedonic etiology. While the precise role of the striatum in regulating nuanced feeding behavior remains to be elucidated, it is clear that it is sensitive to acquired DA defects mediated by the diet. Thus, it is important to develop a more complete understanding of how pathological feeding dysregulates striatal DA homeostasis and whether restoration of DA homeostasis normalizes feeding.

Neuronal insulin signaling is exquisitely sensitive to dietary macronutrient intake [27] and regulates food intake and reward [35]. In particular, *in vivo* evidence suggest a critical role of insulin signaling in cathecolaminergic neurons to control food intake and energy homeostasis [36]. We and others have shown the molecular mechanism by which CNS monoaminergic systems are regulated *via* insulin/Akt signaling, including the trafficking of monoamine transporters [8], [9], [15], [37]. Here, we mechanistically linked HF diet and development of striatal Akt signaling deficiency with depressed monoamine neurotransmission and predict that these defects lead to pathological overeating. This prediction provides the rationale to pharmacologically rescue monoamine signaling in the brain of obese individuals to modulate feeding. These observations, as well as similar findings from others, suggest a link between brain insulin signaling, Akt activation, and monoamine-related behaviors including caloric intake and locomotion. Disruption of brain insulin action (either genetic or acquired) may, therefore, confer risk for and/or underlie “food-abuse” by dysregulation of striatal DA homeostasis.

We demonstrate for the first time that HF feeding quickly leads to striatal insulin resistance and decreased Akt function (Fig. 1). HF diet also lead to reduced DA clearance (Fig. 2) and DAT plasma membrane expression (Fig. 3), and viral restoration of Akt signaling in dopaminergic substantia nigra-striatal neurons restored DAT surface expression to LF levels (Fig. 6, 7). In order to validate the mechanistic link between Akt and DAT expression/function, we also demonstrated that pharmacologic inhibition of striatal Akt acutely impairs DA efflux (Fig. 4). Thus, our data support a model of diet-induced striatal DA hypofunction resulting from acquired impairment in brain Akt signaling that can be rescued by restoring Akt function in nigra-striatal neurons.

It is clear that a high-fat, high-calorie diet leads to obesity, and reduced DAT surface expression. Locomotor activity is a highly DA dependent function. We show that HF feeding results in diminished AMPH-induced locomotion (Fig. 5). This reduction in locomotor activity is not due to changes in DA receptor signaling since the ability of apomorphine (a DA receptor agonist) to induced locomotion was not significantly different in LF versus HF animals. Our data suggest that HF diet, by impairing DA function, may support the development of DIO with multiple mechanisms, including decreased locomotion in response to stimuli.

Because linking Akt dysfunction to DA hypofunction is fundamental to our model of pathological eating, we sought to validate this mechanism by determining if viral rescue of Akt function could, in turn, rescue DAT expression, function, and DA-associated behaviors. The two main DA projections originate from the substantia nigra to the dorsal striatum, and from the ventral tegmental area to the ventral striatum. We focused on dorsal striatum since the ventral striatum does not appear to underlie the primary motivation for feeding [18]: disruption of DA signaling in the nucleus accumbens does not impair food intake [34]. Injection of a HSV-IRS2 into the cell bodies in the substantia nigra (Fig. 6) of HF animals increased both IRS2 content and phosphorylation of Akt in the cell bodies of DA neurons in the substantia nigra to levels comparable to that of LF animals injected with either control or IRS2 virus (Fig. 6). This intervention rescued DAT cell surface expression, in the dorsal striatum of HF animals to levels observed in LF controls (Fig. 7). These data support our molecular model of a link between striatal Akt deficiency and impaired DA function. This led us to ask if this also rescued DA related behaviors including AMPH-induced locomotion and increased food intake. Viral rescue of Akt and DAT expression restored the locomotor response to AMPH (Fig. 7) and, importantly, reduced caloric intake of rats fed a HF diet to that equivalent of rats fed a LF diet (Fig. 8), thereby reinforcing a molecular link between Akt, DA, locomotion and feeding. As the role of striatal function in feeding behavior begins to emerge, a higher resolution understanding of alterations in DA systems and Akt signaling by HF diets promises new insights into obesity pathogenesis that may yield new therapeutic opportunities.

## Methods

### Animals

All experiments were conducted in accordance with the National Institutes of Health *Guide for the Care and Use of Laboratory Animals* and the Institutional Animal Care and Use Committee guidelines of Vanderbilt University and the University of Texas Health Science Center at San Antonio.

### Diet induced obesity (DIO) model

Male Sprague-Dawley rats were ordered from Charles River (Indianapolis, Indiana) at a body weight range of 275–300 g. Upon arrival to the vivarium, they were housed individually in a facility kept on a 12-hour light cycle, and were given standard rodent chow and water *ad libidum*. All rats were given a diet consisting of 10% fat (LF) (Research Diets D12492 and D12450B, New Brunswick, NJ) for 7 days. After this lead-in period, half of the rats were switched to an isocaloric, nutrient matched high-fat (HF) diet of 60% fat for 28 more days; the other half of the rats were kept on the LF diet for the same amount of time. Both rats and food were weighed daily. Adiposity was determined by magnetic resonance spectrometry (Echo Medical Systems, Houston, TX) as performed earlier [27]. DIO was defined as a 10% increase in body weight in the HF group compared with the LF group [27]. Caloric consumption was calculated by converting the weight of the food consumed to kilocalories using the conversions for the respective diets: 60% fat was 5.24 kcal/gram, and 10% was 3.85 kcal/gram. All experiments were performed in the morning, with the exception of HSCS recordings that began in the morning and ran into the afternoon.

### Tissue preparation

Tissue punches from specific brain regions were collected (dorsal striatum and substantia nigra) and homogenized on ice in buffer containing 20 mM Tris (pH 7.5), 150 mM NaCl, 1 mM EDTA, 1% Triton X-100, 2.5 mM sodium pyrophosphate, 1 mM β-glycerolphosphate, 1 mM Na_3_VO_4_, 1 mg/ml leupeptin and 1 mM PMSF, then spun at 13,000×g for 30 minutes at 4°C. The supernatant was taken, the protein content assessed, and analysis performed.

### Brain slice preparation

Methods are as described by Grueter *et al.* [38]. Rats were decapitated, the brains quickly removed and placed in an ice-cold, low-sodium/high-sucrose dissecting solution (consisting of (in mM): 210 sucrose, 20 NaCl, 2.5 KCl, 1 MgCl_2_, 1.2 NaH_2_PO_4_, 10 glucose, 26 NaHCO_3_). Hemisected (300 µm) coronal brain slices containing the striatum were prepared on a vibratome (Leica VT1000S). Slices were allowed to recover in a submerged holding chamber (37°C) containing oxygenated (95% O_2_, 5% CO_2_) artificial cerebrospinal fluid (aCSF) that contained the following (in mM): 124 NaCl, 4.4 KCl, 2.5 CaCl_2_, 1.3 MgSO_4_, 1 NaH_2_PO_4_, 10 glucose, and 26 NaHCO_3_ for a recovery period of 60 min before beginning experiments. Biotinylation assays were then performed.

### Biotinylation assays

Biotinylation of brain slices was done as previously described [39], with some modifications. Hemisected slices (300 µm) were made as described above and transferred to multiwell submerged chambers containing oxygenated aCSF with EZ-Link™ Sulfo-NHS-SS-Biotin (Thermo Scientific, Rockford, IL) at 1.0 mg/ml on ice, and incubated for 45 minutes, then washed twice for 10 min in aCSF, and finally incubated in aCSF containing glycine (100 mM) for two, 20 min periods. Slices were placed onto dishes on dry ice, and the frozen striatum then was removed and placed into eppendorf tubes. The frozen tissue punches were homogenized in ice-cold homogenization buffer (1% Triton X-100, 2 mM sodium orthovanadate, 2 mM sodium fluoride, 25 mM HEPES, 150 mM NaCl, 10 µg/ml aprotinin, and 10 µg/ml leupeptin, and 100 µM phenylmethylsulfonyl fluoride) and then at centrifuged at 17,000 g at 4°C for 30 min. The supernatant was then added to 500 µL of 0.1% Triton X-100 buffer: 150 mM NaCl, 25 mM HEPES, 2 mM sodium orthovanadate, 2 mM sodium fluoride, 1 µg/ml leupeptin, 1 µg/ml aprotinin, 1 µg/ml pepstatin, 0.5 mM phenylmethylsulfonyl fluoride. The protein concentration of each sample was measured and equal amounts of protein for each sample were incubated with ImmunoPure immobilized streptavidin beads (Pierce) overnight at 4°C under gentle rotation. Beads were washed three times with 0.1% Triton buffer and biotinylated proteins were eluted in 50 µL of 2× Laemmli sample loading buffer at 95°C for 5 min, then cooled to room temperature. Biotinylated and total lysate samples were analyzed by SDS-PAGE and Western blotted with a DAT primary mouse monoclonal antibody (Dr. Roxanne Vaughan, UND) and Na/K ATPase antibody.

### Immunoblotting

For Western blotting, determination of immunoreactivity was conducted according to previously described methods [7], [9]. Briefly, tissue samples were separated by SDS-PAGE, and resolved proteins were transferred to polyvinylidene difluoride (PVDF) membranes (BioRad), which were incubated for 1 hr in blocking buffer (5% BSA and 0.1% Tween20 in Tris-buffered saline). The blots were then incubated with primary antibody overnight at 4°C. The primary antibodies used included: Akt (1∶1000; Cell Signaling Technology; Danvers, MA), phospho-Akt (Thr308) (1∶1000; Cell Signaling Technology; Danvers, MA) Na/K ATPase (1∶450; Dr. Fambrough, Johns Hopkins University; Baltimore, MD), IRS2 (1∶1000; Upstate Technologies; Billerica, MA), and DAT (mouse monoclonal, 1∶500) [40]. All proteins were detected using HRP conjugated secondary antibodies (1∶5000; Santa Cruz Biotechnology, Santa Cruz, CA). After chemiluminescent visualization (ECL-Plus; Amersham; Piscataway, NJ) on Hyper-film ECL film (Amersham), protein band densities were quantified (Scion Image; http://www.scioncorp.com) and normalized to control.

### Viral Injections

At day 25 of the diet, rats were anesthetized with isofluroene inhalation and given 0.5 µl bilateral microinjections of HSV vectors encoding GFP (as a control), or wild-type IRS-2 as described in Russo *et al.* [33] over 5 min into the substantia nigra (A/P −5.3, M/L +/−2.0, D/L −7.8, measured from bregma). After a 5 minute pause, the needle was slowly withdrawn over a 5 minute period. Biochemical and behavioral assays were performed as described above, 3 days after surgery.

### Immunohistochemistry

For immunohistochemical experiments to confirm viral injections, rats were perfused with 4% paraformaldehyde in PBS and the intact brains were removed, postfixed for 24 hours, put into PBS with 20% sucrose overnight, and then sectioned and processed according to previously published protocols [33]. Briefly, sections were incubated in blocking buffer (containing BSA) and then with rabbit antibody to GFP (1∶5000; Abcam Inc.; Cambridge, MA) and DAT (mouse monoclonal antibody 16, 1∶1000). After incubation with secondary fluorophores, immunofluorescence was imaged using a Perkin Elmer UltraView confocal microscope with a Nikon Eclipse 2000-U microscope equipped with a 60× lens with an N.A. of 1.49. Confocal microscopy was done as previously described [41]. Image processing was performed using Image J and Adobe Photoshop®.

### High-speed chronoamperometry (HSCA)

HSCA was conducted using the FAST-12 system (Quanteon; http://www.quanteon.cc) as previously described with some modification [9], [10]. Recording electrode/micropipette assemblies were constructed using a single carbon-fiber (30 µm diameter; Specialty Materials; http://www.specmaterials.com), which was sealed inside fused silica tubing (Schott, North America; http://www.schott.com). The exposed tip of the carbon fiber (150 µm in length) was coated with 5% Nafion (Aldrich Chemical Co.; htpp://www.sigmaaldrich.com; 3–4 coats baked at 200°C for 5 min per coat) to provide a 1000-fold selectivity of DA over its metabolite dihydroxyphenylacetic acid (DOPAC). Under these conditions, microelectrodes displayed linear amperometric responses to 0.5–10 µM DA during *in vitro* calibration in 100 mM phosphate-buffered saline (pH 7.4). Animals were anesthetized with injections of urethane (850 mg/kg, i.p.) and α-chloralose (85 mg/kg, i.p.), fitted with an endotracheal tube to facilitate breathing, and placed into a stereotaxic frame (David Kopf Instruments; http://www.kopfinstruments.com). To locally deliver test compounds (see below) close to the recording site, a glass single or multi-barrel micropipette (FHC; http://www.fh-co.com) was positioned adjacent to the microelectrode using sticky wax (Moyco; http://www.moycotech.com). The center-to-center distance between the microelectrode and the micropipette ejector was 300 µm. For experiments in Figure 2 the microelectrode was filled with DA. The study in Figure 4 used a multibarrel configuration in which barrels contained AMPH (400 µM) or vehicle (aCSF) and a third barrel contained the Akt inhibitor (1 mM). The electrode/micropipette assembly was lowered into the striatum at the following coordinates (in mm from bregma, Paxinos, G. and Watson, C. *The Rat Brain in Stereotaxic Coordinates*, New York, Academic Press, 1998): A/P +1.5; M/L, +/−2.2; D/V, −3.5 to −5.5. The application of solutions was accomplished using a Picospritzer II (General Valve Corporation; http://www.parker.com) in an ejection volume of 100−150 nl (5–25 psi for 0.25–3 s). After ejection of test agents, there is an estimated 10–200-fold dilution caused by diffusion through the extracellular matrix. To record the efflux and clearance of DA at the active electrode, oxidation potentials - consisting of 100-ms pulses of 550 mV, each separated by a 1-s interval during which the resting potential was maintained at 0 mV - were applied with respect to an Ag/AgCl reference electrode implanted into the contralateral superficial cortex. Oxidation and reduction currents were digitally integrated during the last 80 ms of each 100 ms voltage pulse. For each recording session, DA was identified by its reduction/oxidation current ratio: 0.55–0.80. At the conclusion of each experiment, an electrolytic lesion was made to mark the placement of the recording electrode tip. Rats were then decapitated while still anesthetized, and their brains were removed, frozen on dry ice, and stored at −80°C until sectioned (20 µm) for histological verification of electrode location within the striatum. HSCA data were analyzed with GraphPad Prism®.

### Locomotor activity

Locomotor activity was assessed by placing the rat in a 26×61×23 cm high plexiglass chamber located within sound-attenuating cubicles. Horizontal activity was measured with four pairs of infrared photobeams positioned 4 cm above the floor of the chamber. Each beam was placed 15 cm away from the next immediate photobeam and the two extreme photobeams were located 8 cm away from the floor sides. An hour baseline was recorded, rats were given AMPH (1.78 mg/kg, i.p.) and placed immediately back into the chambers to continue recording for 60 minutes. The data was collected in 5 minute periods over each 60 minute test.

## Supporting Information

Figure S1 **HF feeding results in weight gain as well as increased caloric intake and plasma insulin, but not changes in plasma glucose.** The HF-fed rats showed a (A) significant increase in total caloric intake (n = 13/group; *p<0.05 by Student's t-test) and (B) weight gain (n = 13/group; *p<0.05 by Student's t-test) over the 28-day feeding period. On day 28, blood was collected, and plasma glucose and insulin levels were measured. (C) Plasma glucose levels were not significantly different between the two groups (p>0.05; n = 13/group), but (D) insulin levels were (*p<0.05; n = 13/group). All data are represented as mean ± S.E.M.
(TIF)
