# Supplementary figures and images for: Impaired Striatal Akt Signaling Disrupts Dopamine Homeostasis and Increases Feeding
Source: PLoS One. 2011 Sep 28;6(9):e25169. doi: 10.1371/journal.pone.0025169 (PMC3182178; doi:10.1371/journal.pone.0025169)

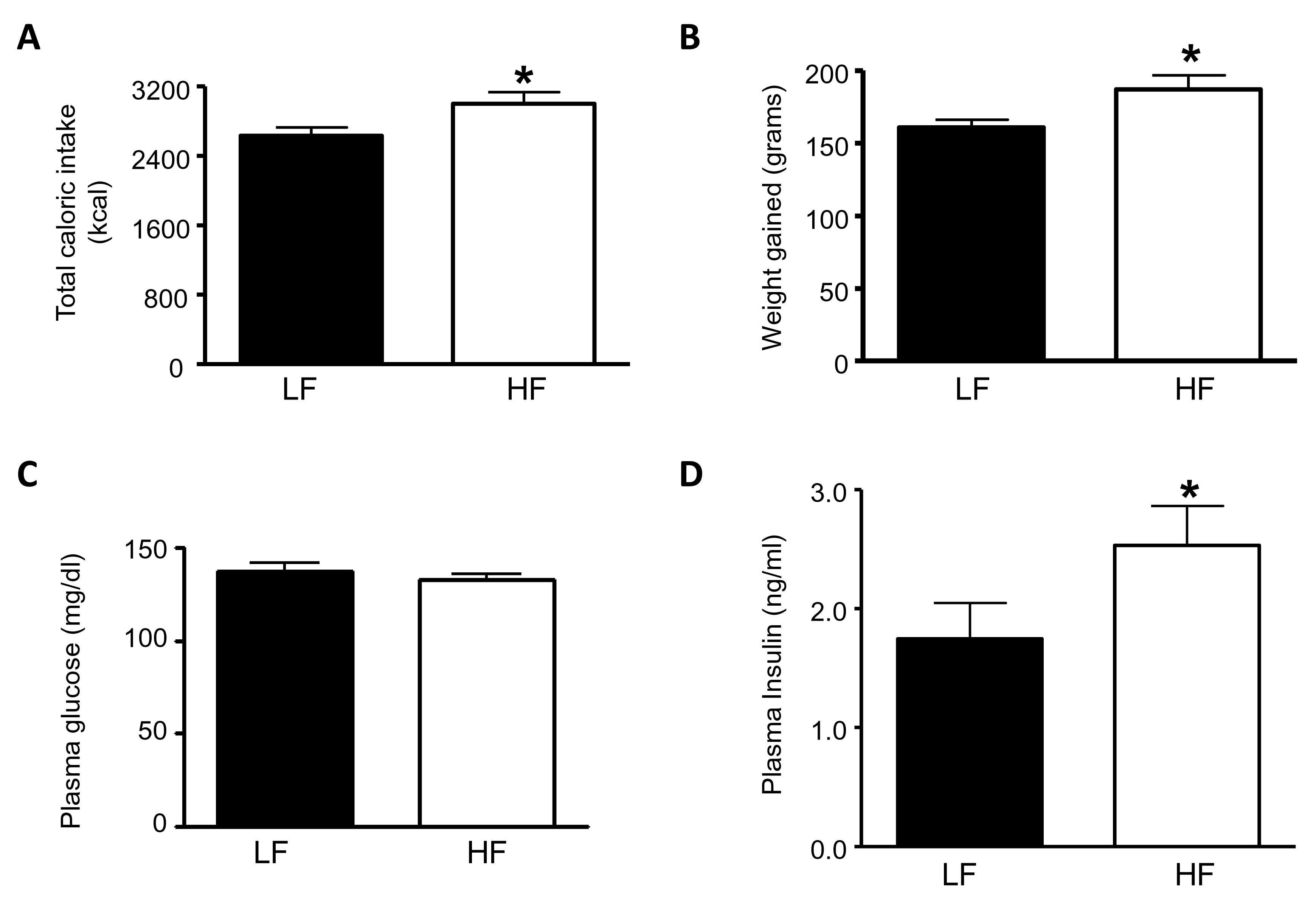

Supplement: Figure S1 — HF feeding results in weight gain as well as increased caloric intake and plasma insulin, but not changes in plasma glucose. The HF-fed rats showed a (A) significant increase in total caloric intake (n = 13/group; *p<0.05 by Student's t-test) and (B) weight gain (n = 13/group; *p<0.05 by Student's t-test) over the 28-day feeding period. On day 28, blood was collected, and plasma glucose and insulin levels were measured. (C) Plasma glucose levels were not significantly different between the two groups (p>0.05; n = 13/group), but (D) insulin levels were (*p<0.05; n = 13/group). All data are represented as mean ± S.E.M. (TIF) [file pone.0025169.s001.tif]
